# Zika in Twitter: Temporal Variations of Locations, Actors, and Concepts

**DOI:** 10.2196/publichealth.6925

**Published:** 2017-04-20

**Authors:** Anthony Stefanidis, Emily Vraga, Georgios Lamprianidis, Jacek Radzikowski, Paul L Delamater, Kathryn H Jacobsen, Dieter Pfoser, Arie Croitoru, Andrew Crooks

**Affiliations:** ^1^ Center for Geospatial Intelligence Department of Geography and Geoinformation Science George Mason University Fairfax, VA United States; ^2^ Department of Communication George Mason University Fairfax, VA United States; ^3^ Department of Global and Community Health George Mason University Fairfax, VA United States; ^4^ Center for Geospatial Intelligence Department of Computational and Data Sciences George Mason University Fairfax, VA United States

**Keywords:** Zika virus, social media, Twitter messaging, geographic information systems

## Abstract

**Background:**

The recent Zika outbreak witnessed the disease evolving from a regional health concern to a global epidemic. During this process, different communities across the globe became involved in Twitter, discussing the disease and key issues associated with it. This paper presents a study of this discussion in Twitter, at the nexus of location, actors, and concepts.

**Objective:**

Our objective in this study was to demonstrate the significance of 3 types of events: location related, actor related, and concept related, for understanding how a public health emergency of international concern plays out in social media, and Twitter in particular. Accordingly, the study contributes to research efforts toward gaining insights on the mechanisms that drive participation, contributions, and interaction in this social media platform during a disease outbreak.

**Methods:**

We collected 6,249,626 tweets referring to the Zika outbreak over a period of 12 weeks early in the outbreak (December 2015 through March 2016). We analyzed this data corpus in terms of its geographical footprint, the actors participating in the discourse, and emerging concepts associated with the issue. Data were visualized and evaluated with spatiotemporal and network analysis tools to capture the evolution of interest on the topic and to reveal connections between locations, actors, and concepts in the form of interaction networks.

**Results:**

The spatiotemporal analysis of Twitter contributions reflects the spread of interest in Zika from its original hotspot in South America to North America and then across the globe. The Centers for Disease Control and World Health Organization had a prominent presence in social media discussions. Tweets about pregnancy and abortion increased as more information about this emerging infectious disease was presented to the public and public figures became involved in this.

**Conclusions:**

The results of this study show the utility of analyzing temporal variations in the analytic triad of locations, actors, and concepts. This contributes to advancing our understanding of social media discourse during a public health emergency of international concern.

## Introduction

The emergence of social media has presented the general public with a novel avenue to disseminate information, exchange views, and network. Given that over 2.3 billion people worldwide are currently active social media users [1], social media play a significant role in communicating news and opinions. When it comes to health communication, in particular [2], social media have been studied to support a broad spectrum of activities: predicting disease outbreaks by monitoring Twitter references to certain terms [3], devising effective communication campaigns [4,5], supporting behavior change interventions [6,7], and tracking the general public’s views on a variety of issues such as vaccination policies [8]. However, the tools for discerning patterns in these social media discussions pertaining to health are still in their formative stages [8-11]. In this paper, we present a study of the recent discourse in Twitter regarding the Zika outbreak to demonstrate the significance of three types of events: (1) geographical events capturing the evolution of the narrative over time and across locations, (2) social media presence events capturing the impact of and interactions over time among key actors, and (3) concept events that capture the emergence and evolution of key concepts that frame this narrative. Combined, these 3 types of events are capturing the evolution and provide valuable insight of this public discourse process.

Studies of social media content for public health issues tend to address one or more of 3 dimensions of these contributions [8-13]:

1. Their geographical dimension, studying the locations of the participating communities,
2. Their social dimension, studying the acting participants (ie actors) in this exchange, and
3. Their linguistic dimension, studying trending patterns of concepts and associations among terms in the social media discourse.

To advance this emerging field of study, we need to improve our understanding of the structure and evolution mechanisms along these 3 dimensions. Our objective in this study was to demonstrate the significance of 3 types of events: location related, actor related, and concept related for understanding how a public health emergency of international concern plays out in social media, and Twitter in particular. To better articulate this approach to studying health discourse in social media, we use the discussion in Twitter related to the Zika virus outbreak of late 2015 and early 2016 as a test case. We present sample results of the analysis of its content along the above-mentioned 3 dimensions. More specifically, we show that:

- The spatiotemporal analysis of data reveals locational events, capturing the progression of the epidemic (and of the discussion about it) from a localized South and Central American issue to a global one
- The analysis of the Twitter announcements and presence of two key public health organizations, namely, the U.S. Centers for Disease Control and Prevention (CDC) and the World Health Organization (WHO), reveals events related to the social media presence of major actors; thereby capturing their gravity relative to the overall structure of the social network of participants over time
- The analysis of the temporal variation of references to certain Zika-related health issues (such as pregnancy and microcephaly) captures concept events, as terms emerge and connect over time, to frame the social media narrative that emerges through this discourse.

We argue that, jointly, events along these 3 dimensions capture the emergence, convergence, and evolution of such health narratives, and support the systematic study of health-related discourse in social media.

## Methods

### Design and Data Collection

Beginning on December 11, 2015, we accessed the Twitter application program interface (API) to collect tweets referring to the “Zika virus” outbreak. We used the GeoSocial Gauge system prototype [14] to collect tweets mentioning the word “Zika” (and all variants of this string, such as #Zika, #ZikaVirus, etc) and the variant “zikv” that is also used at times to refer to the Zika virus. The GeoSocial Gauge system harvests data from Twitter’s streaming API, and retrieves the tweet content as well as accompanying metadata, including information such as user name, timestamp, and location. Earlier studies have shown the suitability of this sample of data for capturing the geographical footprint and the key actors associated with the social media narrative (eg, [15]).

### Summary Data Characteristics

A total of 6,249,626 tweets about Zika were collected during the 12 weeks from December 11, 2015, through March 4, 2016. From among them, 48.16% (3,010,091), had a geographical location associated with them in their metadata, indicating where these tweets were posted from. Among these geolocated tweets, 1.12% were geolocated using coordinates provided directly by the user’s device (eg, tweets posted using a handheld device with GPS location turned on), 2.14% were geolocated from Twitter using user IP information, and the remaining were geolocated through metadata content in the form of toponyms (ie, place names) from the users profile description. Such toponyms were then resolved into the finest available geographical granularity, using the GeoNames geographical database.

**Figure 1:**
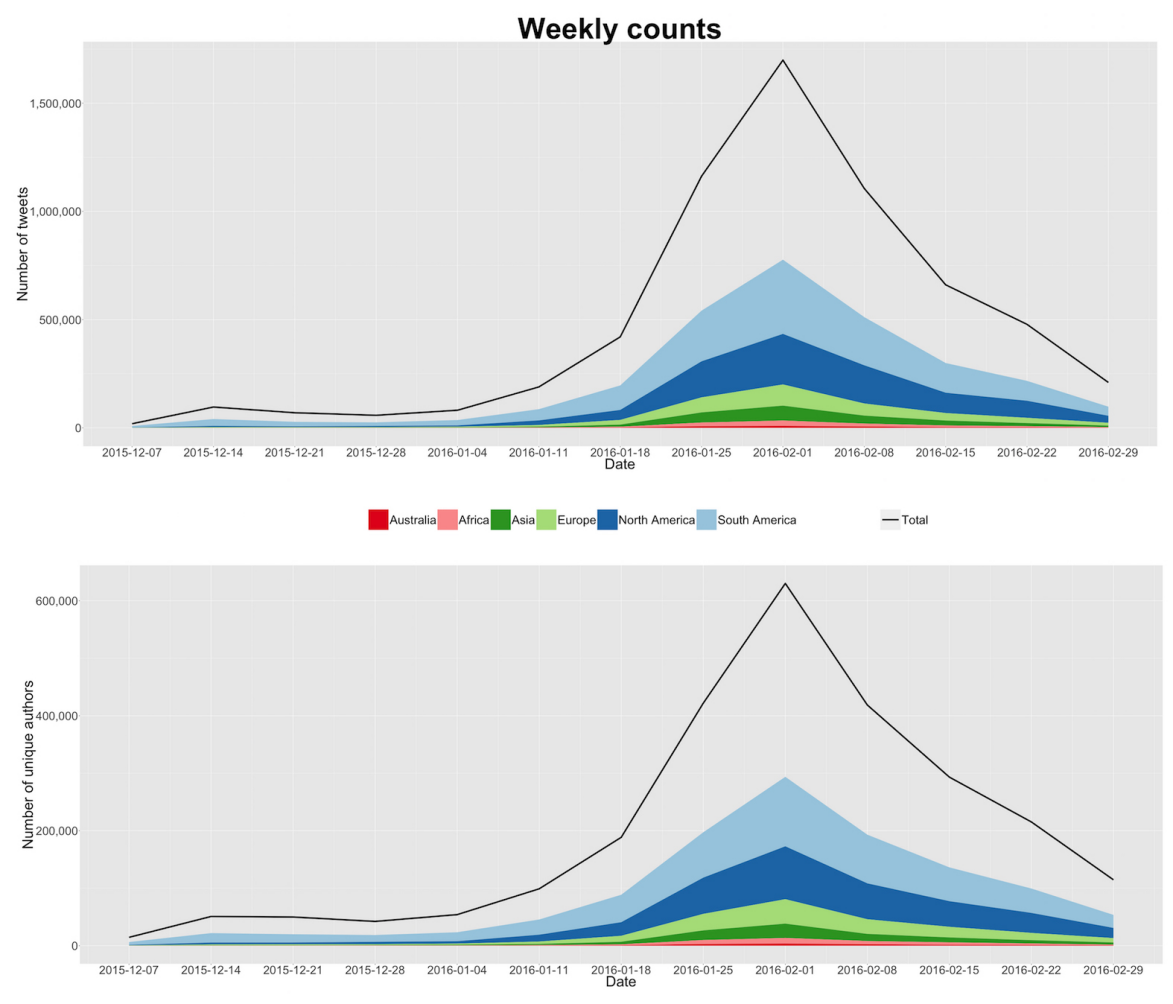
Top: The number of tweets per week. The continuous black line shows the total volume of Twitter traffic mentioning our search terms during our study period. The portion of this traffic that is geolocated is color coded according to the continent of origin. Bottom: The number of unique users contributing to this discussion in the same period, color coded by their continent of origin.

Figure 1 shows the temporal variation of Twitter traffic related to the search terms over our study period, with the data binned in weeks. Traffic grew progressively until it reached its peak during the first week of February, 2016, with a total of 1,698,883 tweets during the first 7 days of the months (882,727 of them geolocated). This corresponds to an average of 126,104 tweets per day during that week. In comparison, during the study period overall there were on average of 74,400 tweets daily. After that peak week, traffic started gradually dropping, approaching prepeak volume patterns. Contributions from South America were prominent throughout the study period. At the peak of Zika traffic (on February 3, 2016), we can identify in our data corpus that 630,142 unique Twitter users who were participating in this discussion, with 293,785 of them being geolocated.

### Social Media Interaction as a Narrative Structure

Since its beginnings almost 50 years ago, the formal study of narrative has crossed many disciplines (such as sociolinguistics, anthropology, and psychology) and applications (eg, medicine, journalism, law); yet it still lacks a clear-cut definition [16]. Broadly defined, the concept of “narrative” is commonly used to refer to storytelling, typically as an orderly sequence of events [17]. Earlier studies addressed narrative in the context of spoken [18] and written [19] expression. A seminal early classification of narrative stories identified 5 distinct types of narratives: (1) official stories from authorities; (2) invented or adapted stories in which the narrative is refined as the public adopts and adapts stories; (3) firsthand stories in which individuals recount their experiences; (4) secondhand stories that are repetition of firsthand stories that have been heard or read; and (5) culturally common stories that reflect common perceptions emerging from the social environment rather than particular individuals [20].

Social media interaction, viewed as narrative, moves beyond individual articulations of past experiences toward encompassing collaboration and greater complexity. The nature of contributions and interactions within social media platforms makes them align most closely with the fifth type of story, culturally common ones [20]. However, all 4 other types of narratives are also present in social media content, as users and authorities alike contribute content, views, and opinions. This makes social media narrative, particularly convoluted, and necessitates novel frameworks for analysis and understanding of them.

**Figure 2:**
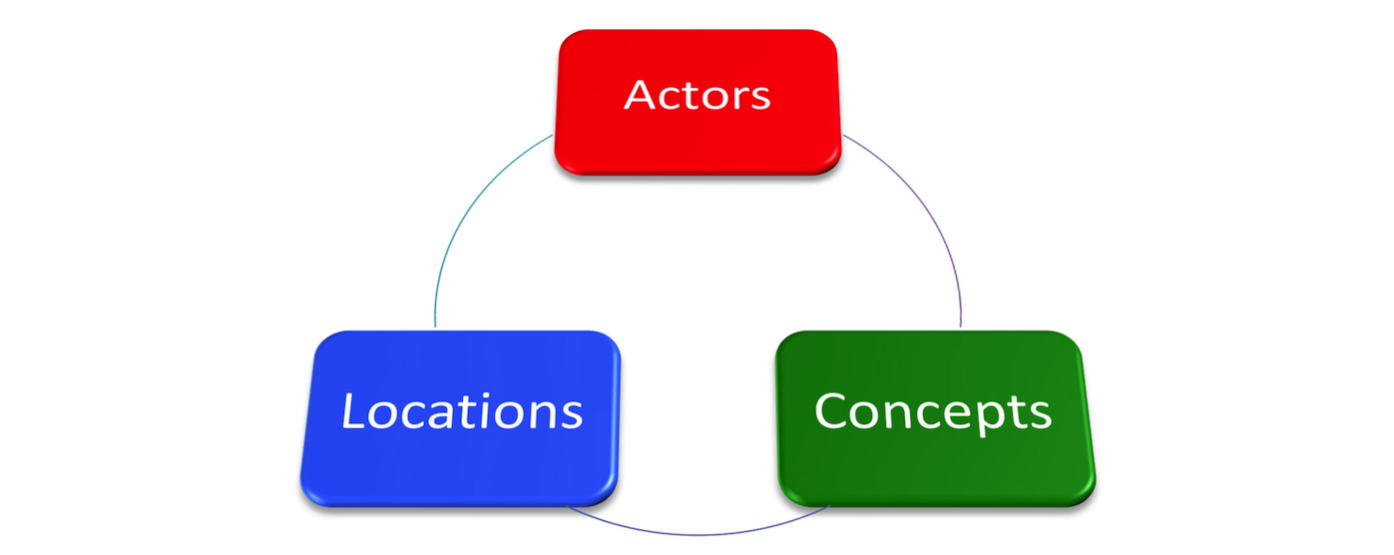
The health narrative triangle.

In a manner comparable with the triadic nature of Meade and Emch’s [21] triangle of human ecology, health narratives in social media have been shown to comprise 3 fundamental and interacting components: locations, actors, and concepts [8]. These 3 components are interconnected, forming a health narrative triangle as shown in Figure 2. Concepts that emerge out of a particular narrative may better engage a specific set of people to participate in that discourse. On the other hand, specific participants may affect with which concepts are introduced and discussed when an overall narrative is formed. As these 3 components encapsulate the overall narrative and represent its foundational elements, here we are focusing on studying its temporal variation through the detection of events along each component, in the form of temporal variations of its defining parameters.

Locations correspond to “where” the narrative is taking place, representing the geographical footprint of the participating communities. Events along this dimension would primarily correspond to changes in the footprint over time, and they can be detected through the application of various spatiotemporal statistical analysis techniques [22].

Actors correspond to “who” participates in and shapes the narrative, reflecting the social dimension of the discourse. They are the contributors to the narrative, with their level of influence dependent upon their overall presence in social media, including their contributions, and the impact of these contributions on the participating community. Events along this dimension can be detected through the analysis of an actor’s contributions and the responses they elicit (ie, in the form of retweets, replies, or mentions), these events can be detected through the analysis of network or trend metrics [23].

Concepts correspond to “what” is part of the narrative, in the form of keywords that are used by the participants in the context of this discourse. They represent the structure of the linguistic dimension of the discourse and encapsulate the associations among terms that are established through crowd interaction. Events along this dimension reveal the emergence of certain terms that expand or redefine the scope of the narrative, and they can be detected through term-trend analysis techniques [24].

### Overview of Data Processing Steps

**Figure 3:**
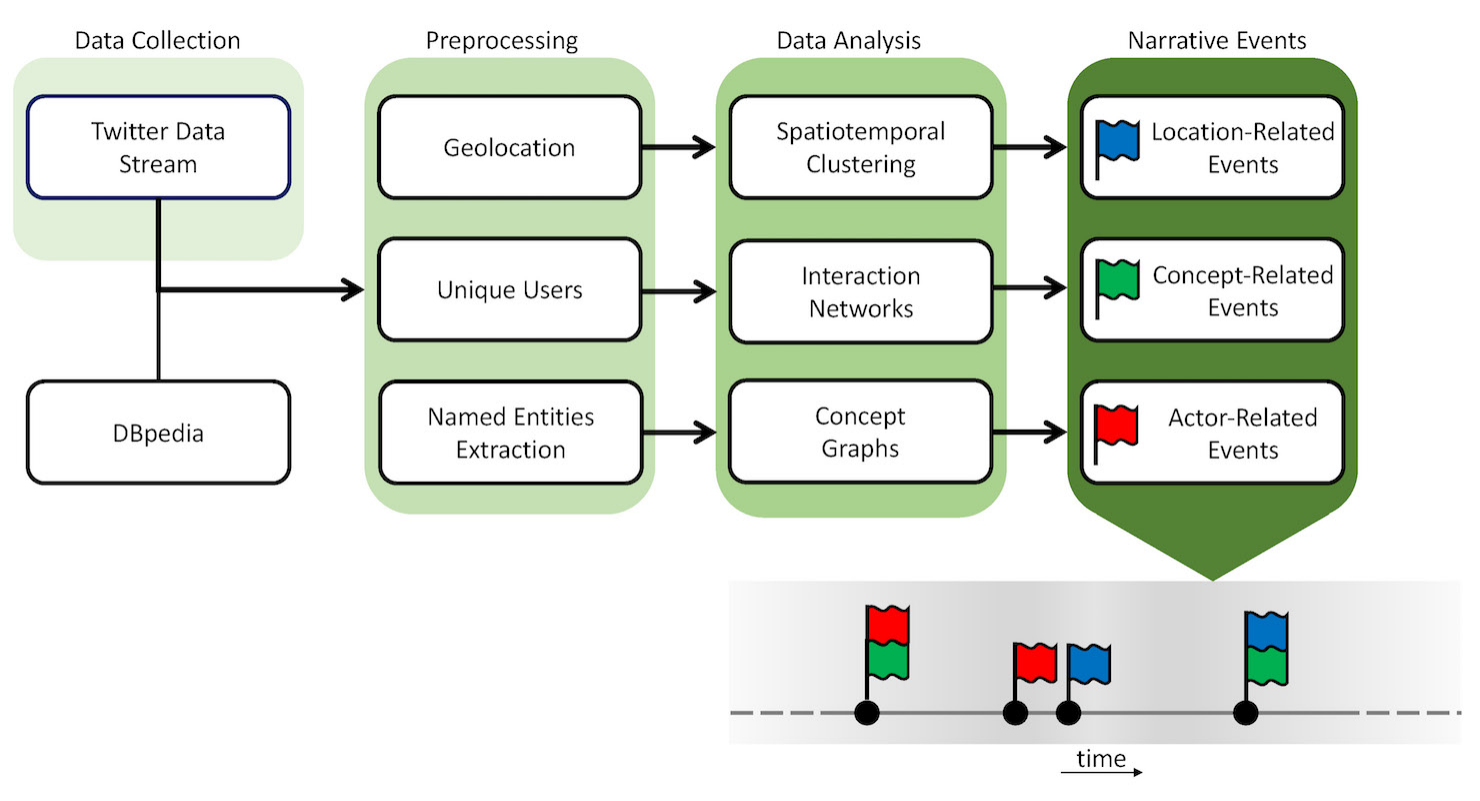
An overview of the Twitter narrative analysis approach, starting with data collection and proceeding with preprocessing and data analysis to identify narrative events, which can be used to build an event storyline.

Our overall approach to process the data comprised a number of automated processes that extract content from streaming Twitter data and process that information to identify corresponding events as shown in Figure 3. The input comprises the Twitter data harvested using Twitter’s API. These data are preprocessed to derive preliminary actor, location, and concept information. Location and actor (ie, user Twitter account) information are derived directly from tweet metadata. Concept information is derived through the identification of named entities. A named entity can be broadly defined in the context of this work as a concept that has a specific definition within an ontology (ie, Zika, or WHO). In our study, the reference ontology is dbpedia [25,26]. These entities are extracted from the content of the harvested tweets and are classified within the corresponding ontology (ie, Zika as disease, and WHO as an organization).

In the next step, we carry out data analysis across these 3 dimensions of the data. Regarding locations, we perform spatiotemporal clustering to identify regions that exhibit higher levels of Twitter traffic clusters compared with the rest of the world. These clusters indicate new communities joining this global discussion. Actor and concept information can be aggregated as corresponding networks. Regarding actors (users), we construct interaction networks of links among them, aggregating interactions in the form of retweets, mentions, and replies within our data corpus. Regarding concepts (named entities), the corresponding graphs capture the co-occurrence of these concepts (named entities) within single tweets. Actor related and concept-related events are derived as changes in the corresponding frequency counts over time. These changes can be monitored either in terms of their absolute values (eg, exceeding thresholds) or the variations of these values (eg, corresponding gradients). When combined, these 3 events serve as the constructs of event storyline summaries of the Twitter narrative (represented in the lower right part of Figure 3) regarding the monitored event (the spread of Zika in this case). These processes are illustrated in more detail under the Results section below.

## Results

### Location-Related Events: Spatiotemporal Patterns

**Figure 4:**
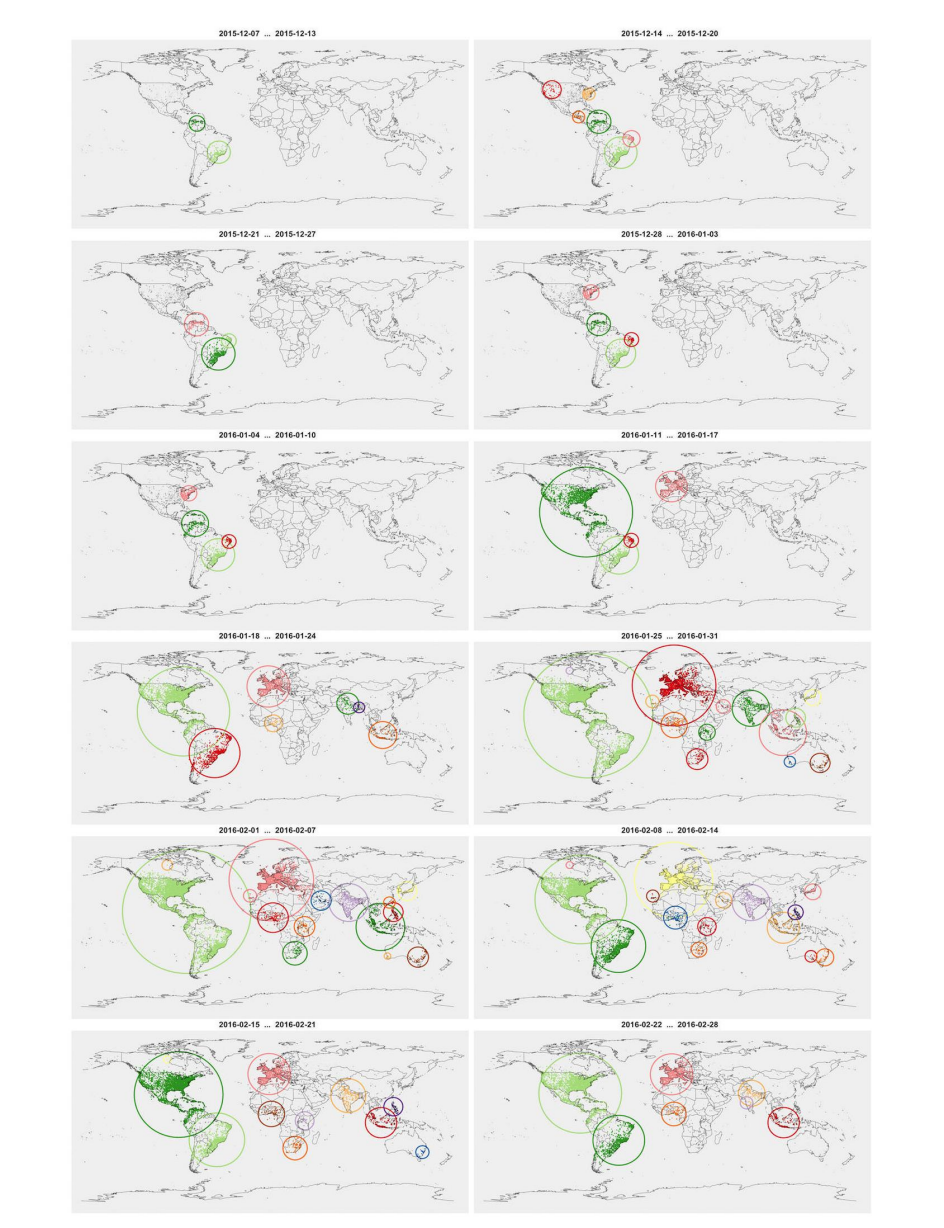
Spatiotemporal participation patterns and identifiable clusters over our 12-week study. The top left panel shows the data during the first week, and time progresses from left to right and from top to bottom toward the 12th week (bottom right).

Figure 4 shows the spatiotemporal evolution of our Twitter data corpus. Each map presents the spatial distribution of geolocated tweets (as colored dots) during the corresponding week. The top left panel shows the data during the first week, and time progresses from left to right and from top to bottom towards the 12th week (bottom right). Together, these 12 panels capture the progressive expansion of the Twitter discussion about Zika from a localized (primarily in the Americas top-left panel) to a global phenomenon during this time period.

Spatial clusters of tweets in a particular week indicate a high level of local participation in this discussion in Twitter. Clusters reveal the regions most engaged in this discourse, and they serve as proxy of local interest for the issue. These clusters therefore are essential constructs of the corresponding health narrative. Discernible changes in these clusters correspond to narrative events, as they reflect instances where participation spread to different communities, driven either by new reported cases or by media coverage.

Several tools automatically detect such clusters in spatial and spatiotemporal data [27-29], primarily focusing on local density and proximity analysis. For our analysis, we used the Density Based Spatial Clustering of Application with Noise (DBSCAN) [30]. DBSCAN is a well-established clustering algorithm that builds on density as a measure for defining and detecting clusters. The algorithm aims to find the maximal set of points that meet a certain density property based on 2 user-defined parameters: a neighborhood radius ε and a minimum number of points *d*. These parameters can be determined through domain knowledge or through a heuristic estimation algorithm (such as the “Automatic Epsilon Calculation” method [31]). DBSCAN offers several distinct advantages, including the ability to distinguish noise in the data, accommodate arbitrary cluster shapes, and perform clustering without previous knowledge or assumptions on the number of clusters. The results of the application of DBSCAN in our datasets are shown in Figure 4. Detected clusters are depicted as colored dots (different colors for different clusters). Additionally, each cluster is delineated by its circular outline, to better signify the corresponding region.

The temporal emergence of these clusters reveals the progressive participation of different communities in this discourse about Zika as it became a global rather than local concern. In early December 2015, there were only two distinct clusters, in Brazil (light green) and the Venezuela/Colombia region (darker green). This is a close match with the locations of reported cases that week [32]. During the following week in mid-December, 2015, new cases reported in Haiti and Puerto Rico, and an intensification of cases occurred in Guatemala and southern Mexico. This corresponds with increased Twitter posts from these areas, with new Twitter clusters in Central America. Additional expansion of global interest continued in the coming weeks, with Europe becoming more involved in Zika social media by week 6 (third row, right panel) and Asia and Africa joining in by week 7 and further intensifying in weeks 9 and 10). In the context of health narrative analysis, events along the location dimension correspond to the distinct instances where additional locations join the Zika discourse, with the virus (and concerns about it) spreading from its original hotbed of South and Central America to communities across the globe.

### Actor-Related Events: Social Media Presence of Health Organizations

To showcase events related to the social dimension of the narrative, we examined the involvement of the CDC and WHO in the Zika narrative. CDC and WHO were selected for our analysis because they were the top two organizations among the identified named entities in our dataset (ahead eg, of Reuters, Google, Monsanto, and the US Congress, which were some of the other top-ranked entities). Furthermore, and arguably not uncorrelated to this fact, these two organizations were clearly the top information disseminators or communicators for health information worldwide, rendering their analysis even more interesting.

**Figure 5:**
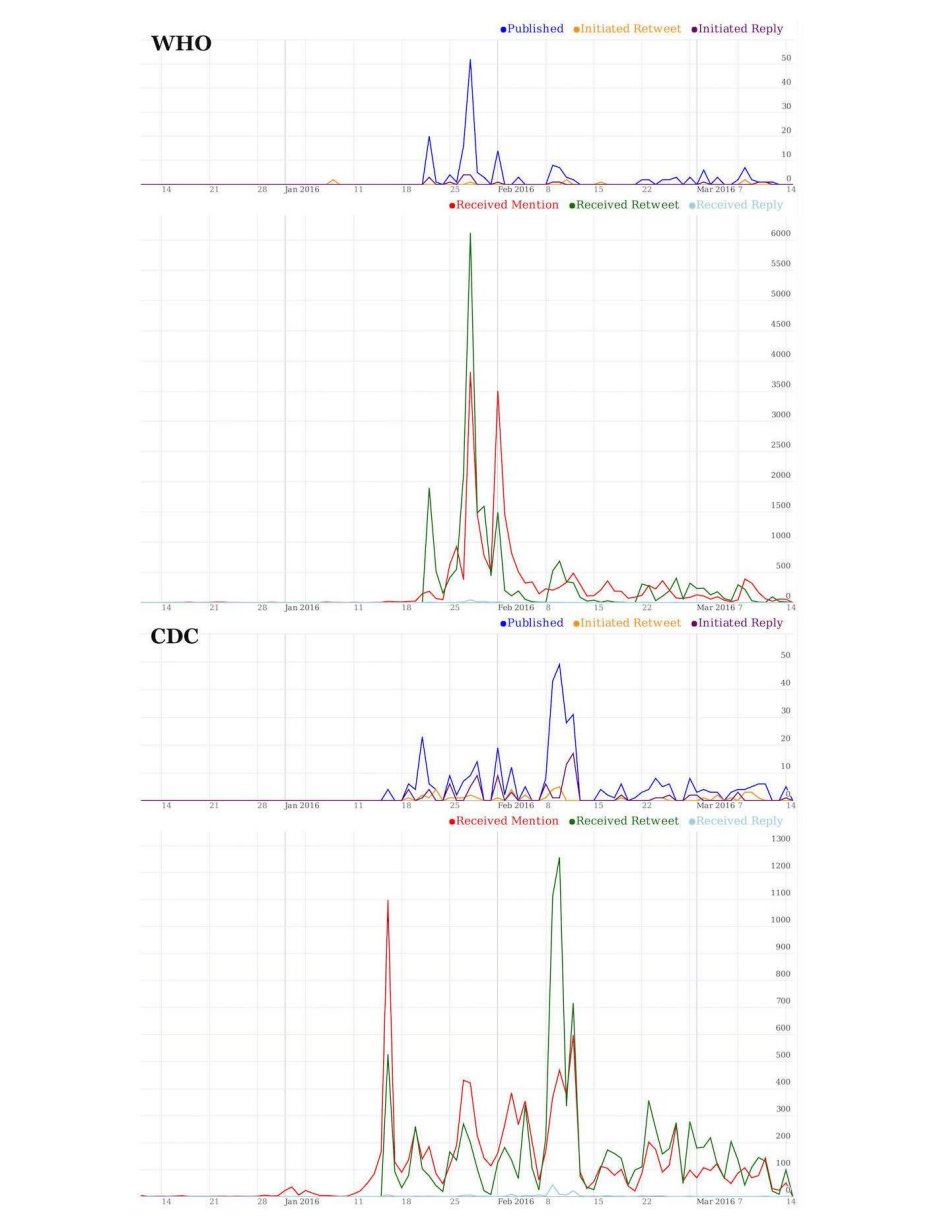
Temporal variations of the social media presence of WHO (top) and CDC (bottom) in the Zika-related discussion in Twitter during our study period. The horizontal shows time, and the vertical numbers of tweets or retweets or mentions as appropriate.

Figure 5 shows the levels of presence in our data corpus of WHO (top) and CDC (bottom). For each organization, the top graph shows activity and the bottom graph depicts impact. All four charts show time along the horizontal axis (with December 2015 at the extreme left and March 2016 at the extreme right). The vertical axis captures metrics of social media presence for these organizations, as follows. The upper part of each chart is a social media activity subchart, expressing how active these organizations were during our study period, and comprises (1) a blue line, showing the daily variations of the number of contributions (original tweets) made by these organizations over time; (2) an orange line, showing how often these organizations retweeted the posts of another user; and (3) a purple line, showing how often these organizations replied to another user. The bottom part of each chart is a social media impact subchart, expressing how well this activity resonated within the Twitter community, and comprises (1) a red line, showing how often these organizations were mentioned in tweets by other users in our data corpus (ie, in the context of Zika); (2) a green line, showing the number of times that a message originally posted by these organizations was retweeted by the general public in our data corpus; and (3) a cyan line, showing how often other users replied directly to these organizations’ posts.

Overall, the level of reply activity is substantially lower than retweeting or mentions. This suggests that Twitter users view these organizations’ accounts as information sources rather than portals for active interaction with them. The vertical axis of both social media activity subcharts spans the range 0 - 50. The maximum number of Zika tweets per day for WHO was 52, while for CDC it was 49. The vertical axes for the other colored charts span the range 0 - 6000 for WHO and 0 - 1500 for CDC. Together, social media activity and impact express the overall social media presence of these organization’s accounts *.*

CDC had higher levels of activity, publishing more original tweets (241 vs 152, respectively, shown in the blue chart), replying to more tweets (93 vs 18, purple lines), and retweeting more (45 vs 9, orange lines) than WHO. In contrast, impact shows a reversal of the activity pattern, with WHO receiving more retweets (22,798 vs 10,935, green lines), more mentions (22,468 vs 10,789, red lines) and more replies (185 vs 170, cyan lines) than CDC. This reflects the global scope of WHO, compared with the primarily US-focused CDC.

The contrast between impact and activity expresses the “amplification potential” of these organizations. Numerically, an expression of this potential can be the “amplification factor,” which can be defined as the ratio between the sum of the 3 impact metrics over the sum of the 3 activity metrics. In our data corpus, the WHO had an amplification factor of 254 compared with only 58 for the CDC. In this particular case, the amplification factor is a direct reflection of followership in Twitter. For this particular disease, the WHO amplification factor is 4.4 times that of CDC’s, which is exactly the same as the ratio of their corresponding followership (3.32 million vs 755,000, respectively at the time of writing).

**Figure 6:**
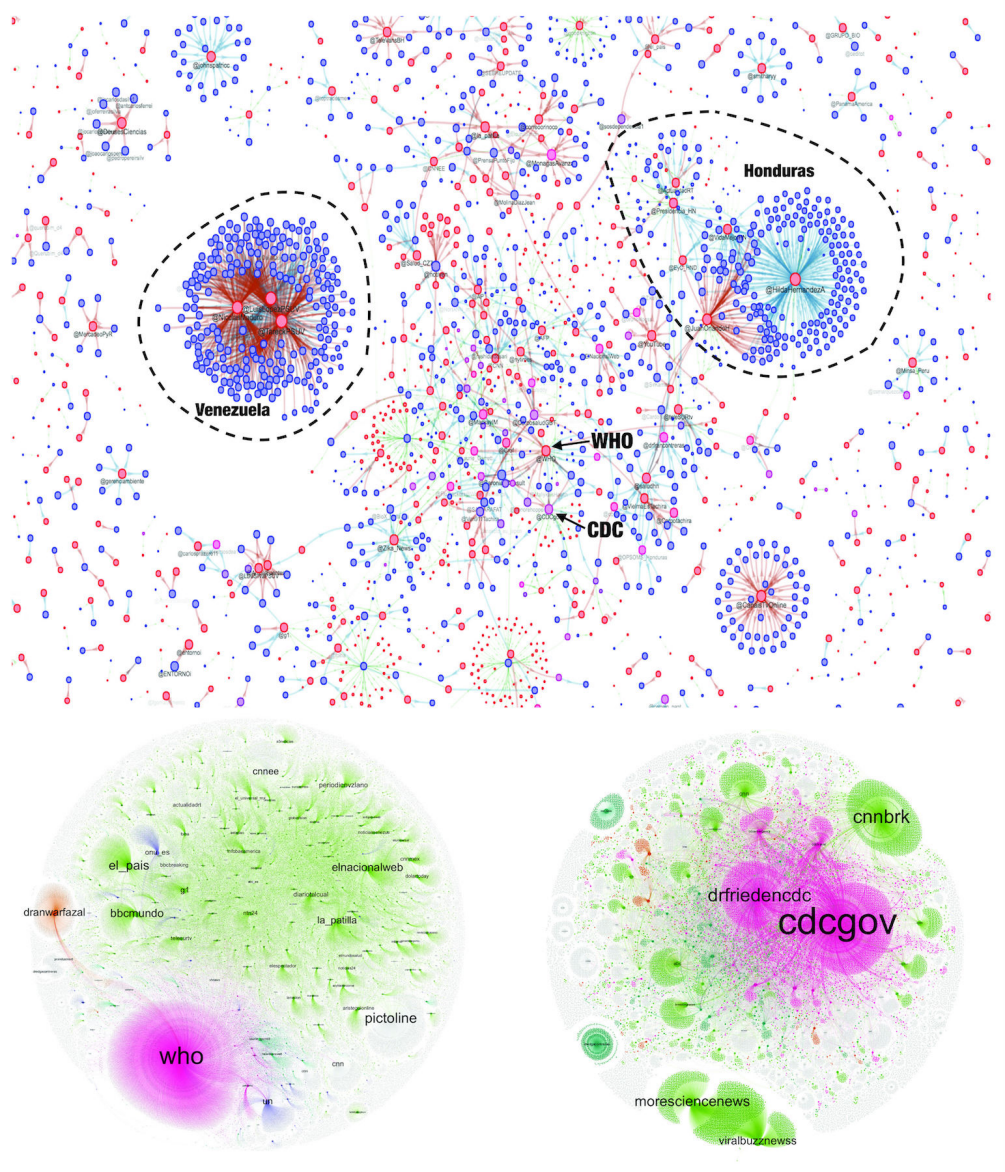
Top: The presence of WHO and CDC in an aggregate interaction network. In this particular figure, we visualized only the top 3,000 connections. Red nodes are originators of information (ie, original authors), blue are consumers (eg, retweeting, mentioning, or replying to these original posts). Bottom: Subsets of the full retweet network pertaining to the WHO (left) and CDC (right), and clusters identified within them. Magenta clusters are centered upon health entities, green upon news organizations, orange upon political entities.

The footprints of these organizations in the full Twitter community can be illustrated with “interaction networks” that capture the aggregate connections among these nodes. In these retweet-mention-and-reply networks, two nodes are connected each time one retweets, mentions, or replies to the other one. Figure 6 (top) shows the placement of WHO and CDC in the broader interaction network when the top 3000 connections are visualized. The size of the nodes corresponds to node activity. In this figure, two distinct identifiable clusters correspond to political leaders and are geographically oriented. One cluster is formed around Venezuelan political figures such as President Nicolas Maduro (@NicolasMaduro) and Tareck El Aissami (@TareckPSUV), whereas the second is formed around Honduran Minister of Communication Hilda Hernandez (@HildaHernandezA).

Figure 6 (bottom) shows the subsets of the full interaction network related to WHO (left) and CDC (right). WHO is primarily connected through its links through news organizations, such the Spanish newspaper El Pais (@el_pais), the Spanish language branches of BBC and CNN (@bbcmundo and @cnnee), and some Venezuelan news websites such as La Patilla and El Nacional (@la_patilla and @ElNacionalWeb). In contrast, the CDC presence is shared by a number of official accounts, including the primary CDC Twitter account (@CDCgov) and those of CDC Director Frieden (@drfriedencdc) and CDC Travel (@cdctravel). Contrasting this with the above presented observations regarding the amplification factor and followership of the WHO and the CDC suggests that engaging news agencies in a health organization’s social media network footprint tends to bear fruit in terms of message penetration and public outreach. This is consistent with earlier work [8] that demonstrated that news outlets played a substantial role (through news stories) in disseminating health-related information through social media than traditional health organizations’ announcements.

In the context of our analysis, key actor-related narrative events (such as major announcements by official agencies) correspond to discernible instances of growth in organizational impact. When Dr Margaret Chan, Director-General of WHO, announced on January 28, 2016, that the Zika virus is “spreading explosively” [33], and again on February 1, 2016, when WHO issued a statement calling Zika a “Public Health Emergency of International Concern” [34], tweets interacting with WHO spiked. Similar peaks were observed for CDC, after it issued an official health advisory on January 15, 2016 [35], and when it updated its guidelines for screening pregnant women on February 9, 2016 [36].

### Concept-Related Events: Pregnancy and Abortion

In our Zika analysis, key concept events correspond to the discernible instances of notable changes in references to various terms in the context of the social media, Zika-related interaction. As we mentioned above, concepts are extracted as named entities corresponding to dbpedia ontology entries. In order to do so, we used the online TextRazor natural language processing (NLP)-based API to parse the text and extract entities. These entities are classified according to the dbpedia ontology.

**Figure 7:**
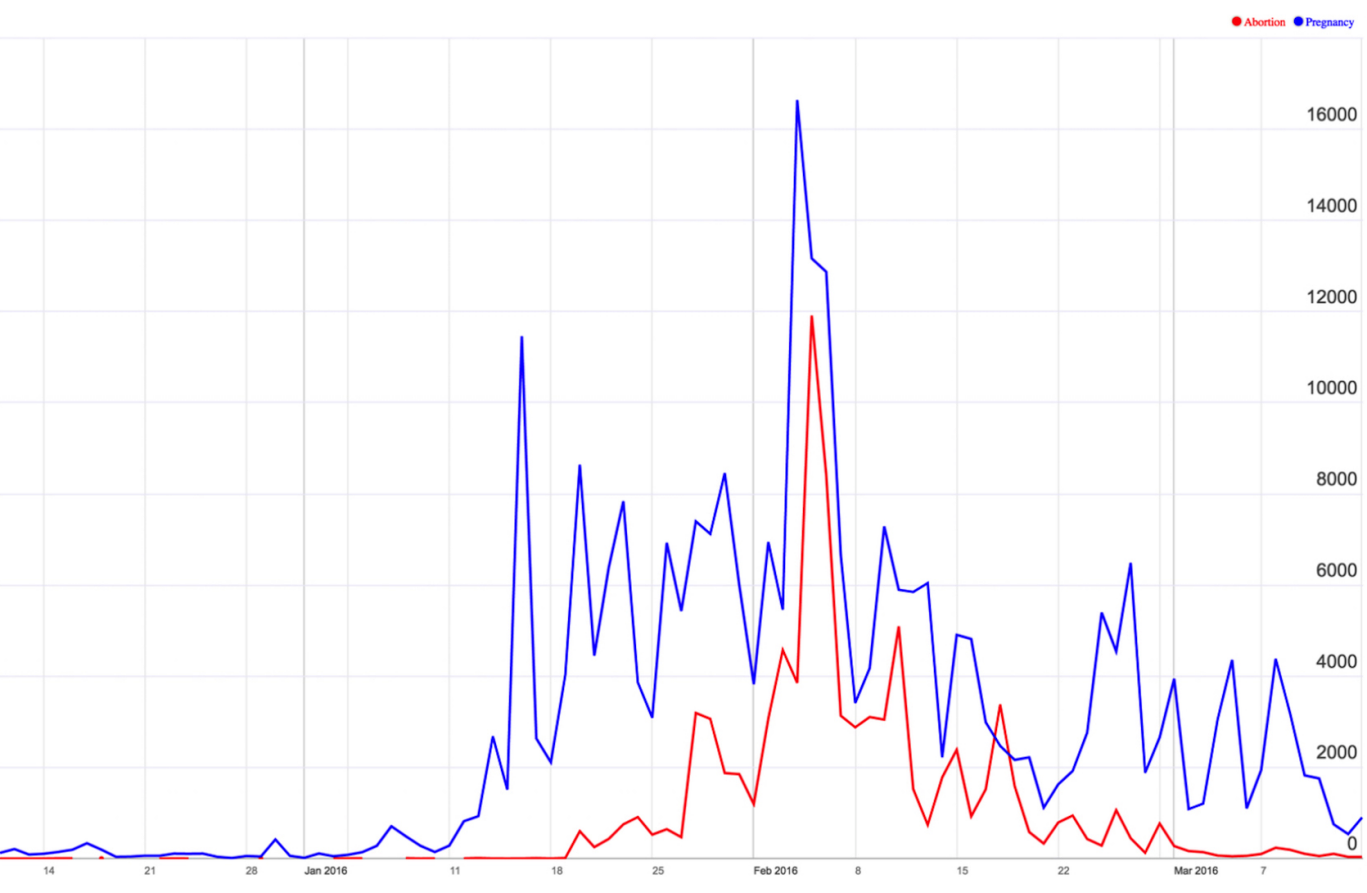
References to pregnancy (blue) and abortion (red) in our data corpus. Horizontal axis: time. Vertical axis: number of mentions.

Figure 7 shows the trending of references to pregnancy and abortion in our data corpus. These two terms were chosen due to their strong emergence during our study period. More specifically, while both terms were rarely encountered during the first weeks of our study, references to pregnancy grew dramatically after January 15, 2016, when the CDC issued its above-referenced interim travel guidance for pregnant women because of growing evidence for the risk of microcephaly in infants born to infected mothers. After that date, the term pregnancy became a key concept associated with the virus, reaching a peak frequency on February 5, 2016, when the CDC offered Zika testing to all pregnant women with potential virus exposure [37]. The term abortion reached a peak on February 6, 2016. Although abortion mentions followed a path overall comparable to pregnancy mentions, they also had an additional off-cycle peak on February 17, 2016, in response to comments by Pope Francis regarding contraception and abortion in the context of Zika [38].

**Figure 8:**
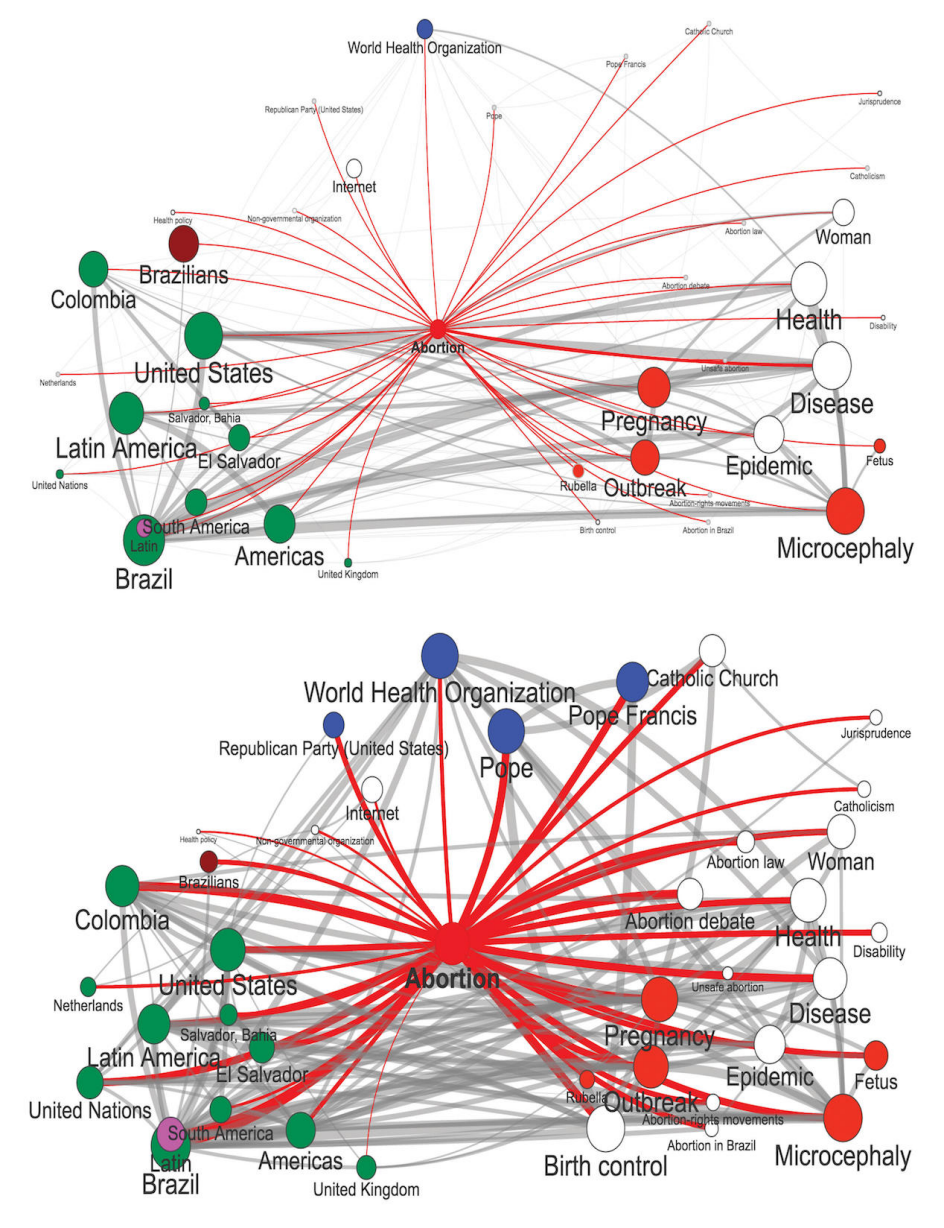
Top: Concept graph for abortion mentions over the 5-day period of 1/1-1/5. Bottom: Concept graph for abortion mentions over the 5-day period of 2/14-2/18.

Figure 8 illustrates the evolution of pregnancy and abortion as key components of the Zika narrative by showing their concept graphs for 2 distinct 4-day periods: January 1 to 4, 2016 (middle) and February 14 to 18, 2016 (bottom). The concept graph captures the terms most frequently encountered in conjunction with the word abortion in our Zika data corpus. The size of the node reflects how frequently a particular term was encountered overall in the data corpus, and the width of the line joining two nodes reflects how frequently these nodes were encountered in the same tweet (expressing a level of mental association of these two terms). Red links are directly related to abortion (radiating outward from that term in the graphs), whereas gray links are connections among these other terms. Abortion was infrequently mentioned in January, 2016. The terms associated with it include ones that match our health narrative triangle: green terms are geographical entities (countries like Brazil and Colombia), red terms are concepts (such as pregnancy and microcephaly) and blue are actors (such as WHO). Mentions of abortion were much more frequent in February, 2016. Although the geographical references remained similar, the concepts had expanded to include terms such as fetus and birth control, and the pool of actors increased to include the Pope and the Catholic Church as key players in the discourse on abortion in the context of Zika. Thus, our triad captures the evolving perceptions of Zika by the general public and the actors that shape this evolution.

### Narrative Storyline Communication

The information captured through the processes outlined above can be viewed as a summarization of the discussion in Twitter regarding the Zika crisis, revealing essential narrative components of the process (eg, in the context of computational narrative models [39-42]). These narrative components are aligned to event storylines as they were addressed in structuralist approaches to narrative formalization [43]. As such, they can inform narrative-driven visualizations [44] of the interaction in social media.

**Figure 9:**
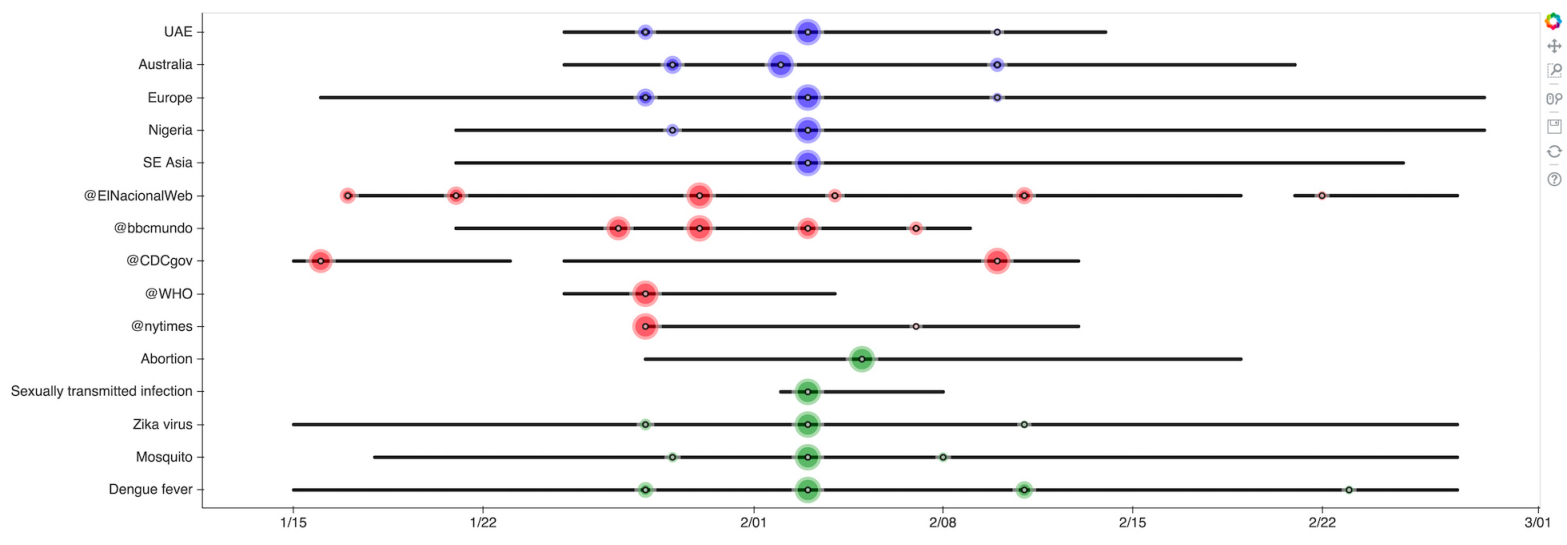
Visualizing a narrative storyline across locations (blue), actors (red), and concepts (green).

In order to demonstrate the potential use of such events for narrative communication, in Figure 9 we present a sample visualization of such summaries, as they were captured through our analysis. We show storylines for representative subsets from each of the 3 dimensions of the narrative, namely 5 locations (blue), actors (red), and concepts (green) over a period of 6 weeks. In this visualization, horizontal black lines indicate continuous activity regarding the corresponding entry, lack of them indicates that a particular topic was below a threshold, and colored disks indicate activity events. The size of the disk is proportional to the magnitude of the event relative to the overall activity. In this manner, we are able to capture both global events (eg, the spikes associated with February 3, 2016, when traffic reached a maximum) as well as localized events, for example, the various spikes before or after that date.

## Discussion

### Principal Findings

This study analyzed how the public engaged with the Zika outbreak of 2015-2016 via social media, from its initial outbreak in South America to its global spread. Our analysis showed how the variations in locations, actors, and concepts involved in Twitter traffic reflect how the health narrative changed as Zika went from being seen as a relatively benign, regional health concern to a global epidemic.

Our findings show how location plays a central role delineating various discrete conversations about Zika on Twitter. The unfolding discussion closely matched maps of the spread of the disease across the globe and expanded news coverage of the emerging infectious disease event. Geographical cohesion is also observed in the clusters of relationships created by retweeting behaviors, with two of the largest clusters emerging from government and political groups within countries dealing with the Zika spread.

At the same time, some of the actors engaged in this conversation crossed geographical boundaries and engaged new actors in the global discussion. Although initially both CDC and WHO largely existed on the periphery of the conversation, we showed how they were able to drive substantial traffic on Twitter when making official announcements.

The combination of geographical clusters and key actors both contribute and respond to the concepts that defined the Zika outbreak. Discussions about pregnancy and abortion as they related to Zika corresponded with public releases from health officials. The Catholic Church also entered into this narrative framework following Pope Francis’s remarks about contraception in response to Zika, in part because of the largely Catholic populations in highly affected areas of the Americas.

The study showed how different populations engaged in the discourse and how actors shape and reshape the discussion and the concepts that the public associates with this public health emergency. Advancing our capabilities to study these temporal variations across these 3 dimensions brings us one step closer to deciphering the complex mechanisms through which the public participates in the discourse. Further work should therefore explore how health campaigns can better use the power of social media for information dissemination campaigns that better align with the dynamic changes of the public health emergency concerns.

## Abbreviations

API: application program interface
CDC: Centers for Disease Control
DBSCAN: Density Based Spatial Clustering of Application with Noise
WHO: World Health Organization
